# Gait Activity Classification on Unbalanced Data from Inertial Sensors Using Shallow and Deep Learning

**DOI:** 10.3390/s20174756

**Published:** 2020-08-23

**Authors:** Irvin Hussein Lopez-Nava, Luis M. Valentín-Coronado, Matias Garcia-Constantino, Jesus Favela

**Affiliations:** 1 Consejo Nacional de Ciencia y Tecnología, Ciudad de México 03940, Mexico; luismvc@cio.mx; 2 Department of Computer Science, Centro de Investigación Científica y de Investigación Superior de Ensenada, Ensenada 22860, Mexico; favela@cicese.mx; 3 Centro de Investigaciones en Óptica, Aguascalientes 20200, Mexico; 4 School of Computing, Ulster University, Jordanstown BT37 0QB, UK; m.garcia-constantino@ulster.ac.uk

**Keywords:** activity recognition, human gait, gait activities, gait classification, inertial sensors

## Abstract

Activity recognition is one of the most active areas of research in ubiquitous computing. In particular, gait activity recognition is useful to identify various risk factors in people’s health that are directly related to their physical activity. One of the issues in activity recognition, and gait in particular, is that often datasets are unbalanced (i.e., the distribution of classes is not uniform), and due to this disparity, the models tend to categorize into the class with more instances. In the present study, two methods for classifying gait activities using accelerometer and gyroscope data from a large-scale public dataset were evaluated and compared. The gait activities in this dataset are: (i) going down an incline, (ii) going up an incline, (iii) walking on level ground, (iv) going down stairs, and (v) going up stairs. The proposed methods are based on conventional (shallow) and deep learning techniques. In addition, data were evaluated from three data treatments: original unbalanced data, sampled data, and augmented data. The latter was based on the generation of synthetic data according to segmented gait data. The best results were obtained with classifiers built with augmented data, with F-measure results of 0.812 (σ = 0.078) for the shallow learning approach, and of 0.927 (σ = 0.033) for the deep learning approach. In addition, the data augmentation strategy proposed to deal with the unbalanced problem resulted in increased classification performance using both techniques.

## 1. Introduction

Activity recognition is one of the most active areas of research in ubiquitous computing with applications in behavior change, risk prediction and early diagnosis [1]. Some of its main challenges include [2]: (i) the fact that human activities, especially human movement, present high intra-and inter-subject variability, and (ii) the daily dynamics of people in complex environments. Most of the works on activity recognition have started investigating from a general outlook, i.e., they focus on classifying activities that are very different from each other, such as walking vs. drinking coffee, instead of a particular, such as walking vs. running [3]. However, due to the granularity of each type of activity, it is necessary to study each of them separately, e.g., gait activities, sleep phases, sedentary activities etc.

Gait activities are directly related to people’s ambulation, deriving the classification: (i) by the speed with which people move, e.g., walking, jogging, running; or (ii) by the surface on which people move, e.g., walking on flat surfaces, walking on stairs, or walking on inclines. According to the type of gait activities to be studied, data gathered from different sensors might be required to classify them. To classify the first group (by movement speed), an accelerometer may be sufficient since it measures the change in speed with respect to time [4], even for different populations like children [5] or older adults [6]. However, when the speed is rather constant, having relatively low variability, the challenge is even greater for activities of the second group (by surface walked on).

Recognizing gait activities is useful to identify various risk factors in people, with one of the main ones being fall prevention. Falls are a leading cause of injury among older adults and most often occur during walking and stair negotiation [7]. Another risk factor is physical inactivity, which according to the WHO is now identified as the fourth leading risk factor for global mortality (World Health Organization: https://www.who.int/). The average healthy adult needs to be active for at least 30 min per day or to walk a minimum of 10,000 steps per day. In some studies, the adult population has been found to walk between 6000 to 9000 steps per day [8,9]. Therefore, it is important to monitor the physical activity of people daily, including gait activities.

Currently, conventional (shallow) machine learning methods have been applied to classify gait activities using statistical features extracted from time-series of different sources, such as inertial sensors. However, the models usually tend to fit the data from each study, as well as the features used to build them, and when the models are tested with a larger dataset, the results cannot be replicated [10]. Therefore, the use of deep learning methods has gained popularity, mainly because they extract a large set of features automatically from different types of data [11]. These methods have been particularly successful for image processing, and thus, one approach has been to transform one-dimensional signals to images, using the features extracted from the images to train their models. Deep learning techniques have achieved great success in many challenging research areas, such as image recognition, Natural Language Processing, and activity recognition [12].

In the recognition of activities and particularly in gait analysis, datasets are usually unbalanced, i.e., there is an unequal number of instances for different classes, in this case, more data from one activity than from the others. One of the reasons for this class unbalance is the fact that the activities performed in naturalistic conditions depend on the daily routine of the people and even their environment, e.g., walking on level ground is the most common activity among people, as opposed to walking stairs [13]. Due to this disparity, the classification models tend to categorize into the class with more instances, the majority class, while at the same time giving the false sense of a highly accurate model [14]. Data augmentation techniques have been proposed in other fields to deal with this problem, being mostly used to balance the number of instances per class.

In the present study, two methods for classifying gait activities using accelerometer and gyroscope data are presented. The proposal is illustrated using a large-scale public dataset and comparing shallow and deep learning techniques. The first method is based in a previous work [15], in which a method for classifying gait activities based on shallow algorithms is proposed with the aim of evaluating its performance with a larger dataset. Additionally, to take advantage of the substantial amount of data, a second method for classifying activities based on a deep learning approach is proposed. Finally, two data treatments to deal with unbalanced data are also proposed in this work. The remaining of the paper is organized as follows. In Section 2 most related work is summarized. Section 3 and Section 4 detail the methods proposed for recognizing gait activities and their evaluation, respectively. Finally, the conclusions and future directions are presented in Section 5.

## 2. Related Work

The recognition of gait activities has been studied from two machine learning approaches: (i) shallow or traditional learning [16,17,18,19,20], and (ii) deep learning [21,22,23,24,25]. As an example of the first category, in [16], accelerometer and gyroscope data were used to classify six gait actions: sitting, standing, lying, walking, running, and cycling. A smartphone was placed on the thigh and an Inertial Measurement Unit (IMU) on the chests of 24 subjects. Time and frequency-domain features were extracted from the segmented signals (15 s) and classified using different algorithms, including C4.5, Classification and Regression Trees, Support Vector Machines (SVM), Multi-Layer Perceptron, and Naive Bayes (NB). Regarding the second category, in [21] the authors propose a one-dimensional (1D) Convolutional Neural Network (CNN) -based method for classifying walking, jogging, and running, by using accelerometer data collected from the smartphones of five subjects. The acceleration data were combined in a vector magnitude and segmented in windows of 10 and 20 s. The performance of the 1D CNN-based method showed 92.71% of accuracy.

In the previously mentioned studies [16,21], the authors collected data from some subjects and used it to evaluate their proposed methods. There are also studies that have collected a large-scale dataset of gait activities from hundreds of people [17,18,19,20,22,23,24,25]. One of these datasets is the OU-ISIR gait dataset, which is described in Section 3.1. This dataset has been used for people identification, and classification of gender, age groups, and gait activities. Works that use this dataset to evaluate their proposed methods, based on shallow learning, are described below.

In [17], features were extracted from acceleration and angular velocity data using a set of gait dynamics information from the sequences of participants’ movement in the lateral view. During the training phase, gait dynamics are approximated using Radial Basis Function neural networks. In the recognition phase, a set of dynamical estimators are generated for the training gait patterns. Recognition errors are produced by comparing the set of dynamical estimators with test gait patterns. The correct classification rate in [17] is 83.3%.

An approach for gait authentication of accelerometer data based on higher order statistics is presented in [18]. Feature extraction is performed using high order cumulants on acceleration data to produce a feature vector of cumulant coefficients. The approach combines feature-level and sensor-level fusion on multichannel and multisensor data. They reported Equal Error Rate (EER) results of 6% to 12%, indicating a reliable performance of the approach considering the number of participants and the location of the sensors.

In [19], an orientation invariant gait-matching algorithm based on the Kabsch alignment is presented for gait authentication. The approach is comprised of methods for splitting cycles, aligning orientation and comparing gait signals. The authors mention that their approach does not require training or learning steps to estimate parameters. The inputs for the gait matching algorithm are sensor data in the form of two time series, each corresponding to the reference and to the test signals respectively. The reported accuracy results estimated from ROC curves are above 90% at 5% false alarm.

In the work presented by [20], the performance of two different approaches (Self-Organizing Maps (SOMs) and Fuzzy C-Means (FCM)) are explored. The focus of their work is to cluster healthy participants divided into age groups. Kinematic features were extracted from Average Cycle Time and cadence. The results reported indicate that better results were obtained with FCM considering that a participant can be part of many clusters. It is also mentioned that the SOM approach does not require defining the number of clusters, which could be useful in cases where there is limited information about a dataset.

Deep learning based approaches applied for the task of gait recognition on the OU-ISIR inertial gait activities dataset obtained much better results than conventional (shallow) approaches. The success of deep learning could depend not only on mathematics but also on physics [22]. In this study, the authors argue that when the statistical process generating the data is of a certain hierarchical form prevalent in physics and machine learning, a deep neural network can be more efficient than a shallow one. This satisfactory performance of deep learning is consistent with results obtained from the data analysis of different types of data, like images or text. The most relevant studies using deep learning based approaches are detailed below.

The approach presented by [23] uses multi-region size CNN for gait recognition from accelerometer and gyroscope data. In this case, a pre-trained CNN for gait feature extraction from a large sensor dataset was used for training and for encoding gait features of another set of participants to be classified using SVM. The experiments carried out were for finding the most adequate parameters for CNN models, and for pre-trained CNN model evaluation. It is reported that their best CNN models applied on the OU-ISIR inertial gait activities dataset provide an accuracy of 96.84% and EER of 10.43%, and that using only a subset of the OU-ISIR dataset their approach can obtain an accuracy of up to (95.53 ± 0.82)% and EER of (11.60 ± 0.98%).

In [24], an end-to-end multi-task and fusion CNN approach that uses raw inertial data as input is presented for gait recognition. From the input raw data, the model learns features that maximize accuracy on the target task via backpropagation. The gait recognition experiments were: (i) sensor position, (ii) single task with individual sensors, (iii) multi-task with individual sensors, (iv) selection of the fusion position, (v) single task with fusion, and (iv) multi-task with fusion. The results reported present an improvement of accuracy (from 83.8% to 94.8%) and the authentication EER (from 5.6 to 1.1).

Another deep learning approach for age and gender recognition from a single inertial sensor is presented in [25], where features are extracted using convolutional feature extraction. The approach performed 10 trials of inter-subject Monte Carlo cross-validation. For the age recognition two classes were considered (teen (age < 20) and adults (age ≥ 20)), and for each trial 70% of the participants were randomly selected for training and the remaining 30% was used for testing. Two experiments were carried out for gender recognition: (i) all participants trained in the same network, and (ii) two separate networks were used for teens and adults respectively. The reported results in [25] were: (i) accuracy for age recognition of 88.6% ± 2.4%, (ii) averaged accuracy for gender recognition of 88.6% ± 2.5%, and (iii) averaged accuracy for the age of 73.9% ± 2.8%.

In contrast to previous work, two important aspects of the present study can be highlighted: (i) two methods for classifying gait activities from the OU-ISIR gait dataset were evaluated and compared, one based on shallow learning (see Section 3.3.1), and the other based on deep learning (see Section 3.3.2); and (ii) the use of data treatments for balancing the dataset, the first based on subsampling, and the other using augmentation techniques to generate synthetic data (see Section 3.2).

## 3. Methods

Human activity recognition using data from inertial sensors is commonly carried out through a process that begins with data acquisition and ends with the classification. In most cases, researchers collect data and propose methods according to this type of data, by processing the signals (the so-called time-series) based on filters designed to the conditions of their setups, as well as adjusting the parameters of the classification models to this data. Regarding the classification step, the most used approach is supervised classification, which requires labeled data and a set of features that best describe the signals obtained with inertial sensors. In this sense, statistical features have been widely used since they usually describe how activities are performed according to the type of signal used, such as raw data, e.g., linear acceleration, or high-level data, e.g., orientation. On the other hand, recent studies have focused on extracting a large number of features by changing the representation of the signals to the image domain in order to take advantage of deep learning techniques.

In previous work [15], a method for classifying gait activities was proposed for a specific setting. Acceleration data was collected from seven young subjects (24–35 years), who performed five gait activities: (i) going down an incline, (ii) going up an incline, (iii) walking on level ground, (iv) going down stairs, and (v) going up stairs, all at a speed comfortable to them. All subjects were asked to carry a smartphone in their right hand, while an inertial sensor was firmly attached to their right ankle using an elastic band. All subjects declared not having any mobility impairment in their limbs. The environments to carry out the activities were the same for all subjects, including both indoors and outdoors.

To evaluate the performance and robustness of the proposed method, a dataset that includes the same gait activities and the same type of data, but under different experimental conditions, was searched, e.g., greater number of participants, other environments, different position of the sensors, among others. This dataset is presented in the following section. Because the data is unbalanced, three types of data partitions are proposed for treatment, see Section 3.2. The proposed method in [15] was adapted as explained in Section 3.3.1. Additionally, a novel approach for extracting deep-based features to take advantage of a large amount of data available is proposed, as detailed in Section 3.3.2.

### 3.1. OU-ISIR Gait Dataset

The OU-ISIR Gait dataset was collected by the Institute of Scientific and Industrial Research (ISIR) of Osaka University (OU) as part of the GAG2019 challenge (OU-ISIR Wearable Sensor-based Gait Challenge: Age and Gender) [26]. The gait activities considered in the OU-ISIR Gait dataset are listed in [27] as (i) level, (ii) up-slope walk, (iii) down-slope walk, (iv) step up, and (v) step down; note that these activities are similar and correspond to the ones considered in the initial dataset previously described in this section. The distribution of age group and gender was not uniform. In addition, the actions were already segmented by steps.

The dataset was gathered from 745 visitors (388 males and 357 females) of varied ages (from 2 to 78 years) of an exhibition in Japan over five days. In this case, three IMU sensors were used to collect the gait data at a sampling rate of 100 Hz. The IMU sensors include a triaxial accelerometer and a triaxial gyroscope, with dynamic ranges at ±4 [g] and ±500 [deg/s] respectively. For the data collection, the participants wore a waist belt that had three IMU sensors attached at different orientations (left, right and center-back). Some of the constraints of the training dataset were the non-uniformity of age groups, sensor location, and orientation inconsistencies. Only data of the sensor identified as center-back were used in this work.

### 3.2. Data Treatments

**Figure 1:**
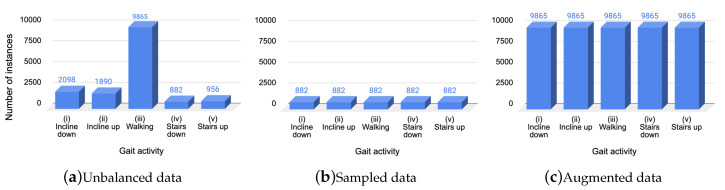
Data treatments: (**a**) original dataset; (**b**) randomly subsampled data from the original dataset; (**c**) augmented data using synthetic data.

As mentioned, the data in the OU-ISIR dataset were unbalanced, which refers to a problem where the number of instances in the dataset for each class label was not balanced. The number of instances for each class was: (i) going down an incline = 2098 (13%), (ii) going up an incline = 1890 (12%), (iii) walking on level ground = 9865 (63%), (iv) going down stairs = 882 (6%), and (v) going up stairs = 956 (6%). We used three data treatments (Figure 1) to train and test the classification models:

1. Using unbalanced data (no alterations in the original dataset), the total instances was 15,691 (see Figure 1a).
2. Balancing data by sampling to the number of instances of the minority class: going down stairs, i.e., the total instances was 4,410 (see Figure 1b).
3. Balancing data by increasing to the number of instances (Section 3.2.1) of the majority class: walking on level ground, i.e., the total instances was 49,325 (see Figure 1c).

#### 3.2.1. Data Augmentation

We applied five techniques to generate synthetic data in order to balance the classes of the dataset used in the present study. These techniques that are commonly used in the field of activity recognition using time-series were analyzed [28,29], implementing those that best fit the data types. The selected techniques were grouped into: (i) Data Modifying the Magnitude of the signal’s (DMM), and (ii) Data Modifying the Frequency of the signals (DMF), and are described below:

1. Scaling (DMM): Multiply all elements of each signal by a random value. The frequency of the signal remained.
2. Jittering (DMM): Multiply a random value to each element of each signal (add noise). The frequency of the signal remained.
3. Smoothing (DMM): Filter each signal using a Hann window with a length of the total−time/10 multiplied by a random value. The frequency of the signal remained.
4. Downsampling (DMF): Random selection of frames and uniform subsampling to reduce the signals. The same rate was applied to all signals. The magnitude of the signals remained.
5. Cutting (DMF): Reduce an equally number of frames (total−time/10 multiplied by a random value) of the edges of the signals. The same rate was applied to all signals. The magnitude of the signals remained.

**Figure 2:**
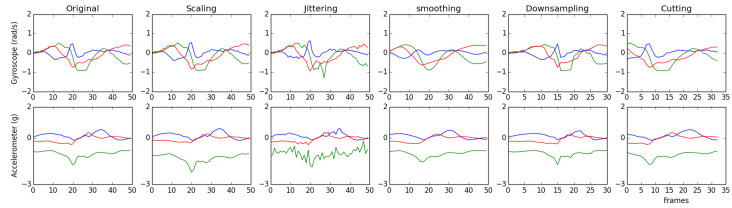
Synthetic data. Top images show gyroscope data while bottom show accelerometer data. From left to right: original signals, synthetic data modifying magnitude of signals (scaling, jittering, and smoothing), and synthetic data modifying frequency of signals (downsampling, and cutting).

The variation factor for the generation of the synthetic data depends on a random value with a Gaussian distribution of mean μ=1 and standard deviation σ=0.2. This parameter is important for the data augmentation since it adjusts to the restrictions of human movement, as well as to the segmentation based on steps. The five techniques were applied iteratively until completing the number of synthetic data required for each class: (i) going down an incline = 7767; (ii) going up an incline = 7975; (iii) walking on level ground = 0; (iv) going down stairs = 8983; and (v) going up stairs = 8909. Figure 2 shows an example of data generated from a segmented step of the walking on level ground activity for gyroscope (top) and accelerometer (bottom) data.

### 3.3. Activity Classification

The data, separated by treatment, were used to classify gait activities applying two approaches. The first of them followed the traditional approach of supervised classification based on statistical features, which describe human movement from the time-series of inertial sensors (see Section 3.3.1). The second approach corresponded to the transformation of the time series in images, from which features were extracted to be used in an artificial neural network (see Section 3.3.2).

#### 3.3.1. Shallow Learning Approach

**Figure 3:**
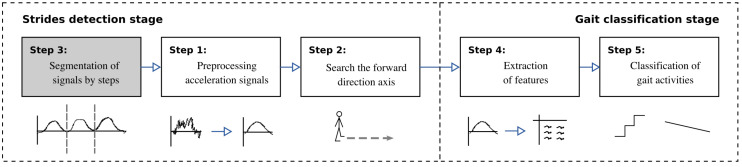
Adapted gait recognition method [15]. Since the data is already segmented the first step (gray) was omitted (Step 3 in [15]).

The adapted method for classifying gait activities is presented in Figure 3 and detailed below. The method was divided into two stages: (i) steps detection, and (ii) gait classification. The main difference from the original method was that the segmentation was already provided in the dataset, so the input data were the segmented steps. When sensors are placed on people’s limbs, it was possible to characterize the strides, or gait cycles, of such a limb. In the dataset considered, the sensor was placed on the torso, making it possible to extract steps. The difference between stride and step was that the former comprised successive contact phases ending by the same foot, while the latter was successive contact phases of opposite feet. Despite this, the proposed method can be used under such circumstances.

Since the data were already segmented the third step (gray) was omitted. In the preprocessing step (first step), a low-pass filter was applied to the accelerometer and gyroscope signals. The purpose of the filter was to avoid artifacts due to movement and to filter the noise in signals. A pseudo-Gaussian smoothing function was then applied to the filtered signals. The first step was applied to the three axes of both types of sensors. Because the signal aligned with the sagittal plane of people was the one that recorded a greater magnitude of acceleration, a search was conducted to identify which of the three acceleration signals has greater power (second step) and it will be labeled as the forward-direction signal FD. In addition, the magnitude vector XYZ was calculated in order to use information of the three signals combined. This vector has been widely used as it is invariant to device rotations.

In the second stage, a supervised classification approach was implemented. We extracted five descriptive features from each step segmented of both accelerometer and gyroscope data: (i) width of the segment, (ii) height of the segment, (iii) mean of the acceleration, (iv) standard deviation of the acceleration, and (v) power of the acceleration. In the last step, the feature vectors are used for building the gait activity classifiers. We selected four inference algorithms because they were appropriate to deal with problems involving unbalanced data. Naive Bayes (NB) classifier is a classification method founded on the Bayes theorem based on the estimated conditional probabilities [30]. C4.5 is a decision tree classifier that builds a binary classification tree; determined by a splitting criterion, features are selected as branching points that separate the two classes [31]. Support Vector Machines (SVM) classify unseen information by deriving selected features and constructing a high dimensional hyperplane to separate the data points into two classes [32]. K-Nearest Neighbors (KNN) is a non-parametric method that does not need any modeling or explicit training phase before the classification process [33].

#### 3.3.2. Deep Learning Approach

In recent years Deep Learning (DL) models have shown outstanding performances to undertake problems in different domains of application such as object recognition, medical diagnosis, self-driving cars, natural language, and predictive forecasting [34]. Convolutional Neural Network (CNN) is one of the most common DL models and has become a state-of-the-art approach in the areas of image processing and computer vision. Inspired by the success of DL and considering that previous works have shown the feasibility of using CNNs using inertial data represented as images for activity recognition [35,36,37], an image-based gait activity recognition using a CNN was implemented.

##### Imaging Time Series

CNN is able to successfully capture the spatial and temporal dependencies in an image through the application of relevant filters. As a first step, the time series is encoded as a Gramian Angular Field (GAF) image (the encoding strategy is based on the work presented in [37]). According to this strategy, to make a GAF matrix, the values $X=\{x_{1},x_{2},\cdots ,x_{n}\}$ of the time series are first scaled into values between [−1,1] by using Equation (1).

(1) $${\tilde{x}}_{i}=\frac{\left(x_{i}-\max\left(X\right)\right)+\left(x_{i}-\min\left(X\right)\right)}{\max(X)-\min(X)}$$

Thus, $\tilde{X}$$=\{{\tilde{x}}_{1},{\tilde{x}}_{2},\ldots {\tilde{x}}_{n}\}$ represents the scaled values. The second step is to represent the scaled data in the polar coordinate system. Equations (2) and (3) are used to generate the polar representation.

(2) $$\begin{array}{cc} \phi & =\arccos\left(\tilde{x}\right),\;\tilde{x}\in \left[-1,1\right] \end{array}$$

(3) $$\begin{array}{cc} r & =\frac{t_{i}}{N},\;\;\;t_{i}\in N \end{array}$$

After transforming the scaled time series into the polar coordinate system, the cosine of the sum of the angles is calculated to make the GAF by using Equation (4).

(4) $$\mathrm{GAF}=cos(\phi_{i}+\phi_{j})$$

**Figure 4:**
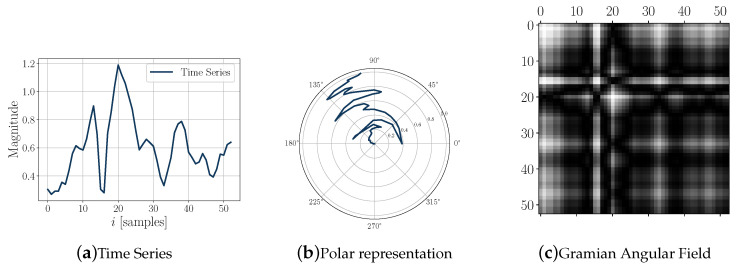
Encoding time-series process: (**a**) angular velocity magnitude time series; (**b**) polar representation; (**c**) GAF image.

Figure 4 shows an example of the time series coding process (in this case the magnitude of the angular velocity components) corresponding to one step.

##### Feature-Based Image

Typically, in terms of colors/channels an image can be represented in two ways: (i) gray-scale (one channel), and (ii) color (three channels: RGB). Considering that a time-series (signal) can be encoded as a one-channel image, the combination of three signals (one per channel) in a feature-based color image is proposed. The proposed signals combination is given by (i) the magnitude vector of acceleration ($Acc_{XYZ}$), (ii) magnitude vector of angular velocity ($Gyr_{XYZ}$), and (iii) forward-direction signal based on the acceleration signal ($Acc_{FD}$).

**Figure 5:**
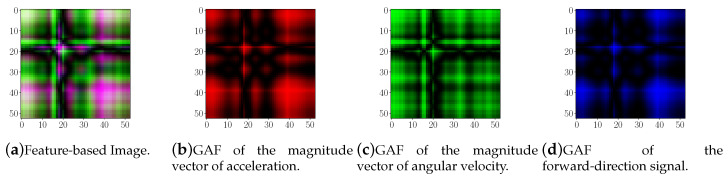
Figure (**a**) shows an example of a feature-based image. Besides, Figures (**b**), (**c**) and (**d**) corresponds to the Red, Green and Blue channels respectively.

In Figure 5 an example of a feature-based image (Figure 5a), as well as its channels (Figure 5b–d) are shown. In this figure each channel is presented according to its corresponding value, for instance, the GAF image of the magnitude vector of acceleration corresponds to the red channel, then, each intensity value of each pixel in the image has a value between 0 and 255, which is shown as a red intensity value.

##### Convolutional Neuronal Network (CNN)

CNN is a type of artificial neural network, most commonly applied to analyze visual imagery. CNN is comprised of units called neurons, which take in a weighted sum of inputs and output an activity level. The activity level is commonly a nonlinear function of the input, frequently just a rectified linear unit where the activity is equal to the input for all positive input and 0 for all non-positive input.

**Figure 6:**
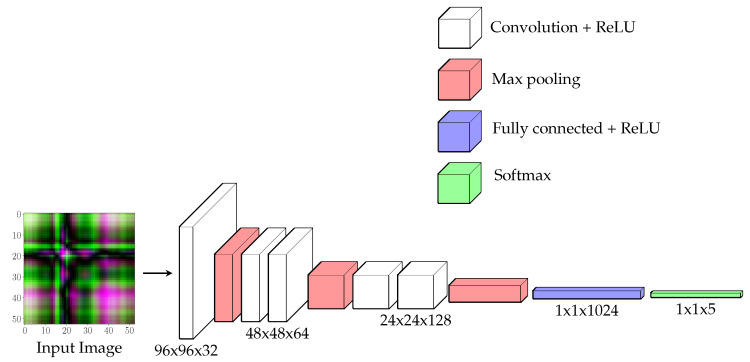
Convolutional Neural Network (CNN) architecture.

The proposed CNN (see Figure 6) is made up of five convolution layers, three max-pooling layers and two fully connected layers. Convolution layers extract features from the input image, preserving relations between pixels; besides, each convolutional layer is followed by a non-linear activation function. The most common choices of activation functions are (i) sigmoid (or logistic), (ii) hyperbolic tangent (tanh), and (iii) a rectified linear (ReLU), defined as simply max(0,x) to ensure that feature maps are always positive. The proposed CNN model uses ReLU, since, as shown in [38], it speeds up the training and sometimes produces more accurate results. Pooling layers are used not only to reduce the number of parameters but also to control overfitting. In particular, a max-pooling operation with a 2×2 kernel was implemented in this work.

## 4. Results and Discussion

In this section, we present the results of applying both approaches, shallow (Section 4.1) and deep (Section 4.2) learning techniques, and the discussion of them (Section 4.3). The results are shown using the data treatments introduced in Section 3.2: original unbalanced dataset, balanced sampled data, and balanced augmented data.

### 4.1. Gait Classification Using Shallow Learning

In order to evaluate the gait classifiers based on statistical features, detailed in Section 3.3.1, feature vectors were divided in two levels: (i) type of sensor (accelerometer data, gyroscope data, and both accelerometer and gyroscope data); and (ii) type of signal (forward-direction signal FD, acceleration magnitude vector XYZ, and both FD and XYZ). Data partition was made using five-fold cross-validation, which indicates that the classifiers were trained with the 80% of the instances and tested with the rest of the instances (20%), repeating the process five times.

#### 4.1.1. Unbalanced Data

Table 1 presents the correctly classified instances rate by using the original dataset, i.e., unbalanced data. Regarding the type of sensor data, combining the data from both shows a better result; there is no significant difference between using accelerometer data or using the gyroscope data separately. Regarding the type of signal, the best result was achieved using the forward direction signal (FD), and combining FD with the magnitude vector XYZ. The best results by treatment for each classifier built from unbalanced data were: 68.2% for Naive Bayes (NB), 72.5% for C4.5, 73.2% for Support Vector Machines (SVM), and 69.7% for K-nearest Neighbors (KNN).

From the best results by classifier, Table 2 shows the individual F-measure by activity. This classification metric provided a single score that balanced both the concerns of precision and recall. The results were similar for all classifiers, however two of the activities presented very low scores for all classifiers too: going down an incline and going up an incline. Despite the fact that the class with the highest number of instances was walking on level ground, there was no clear overfitting with respect to the other classes, since similar or superior results were obtained for activities that involve the use of stairs; this trend could be best distinguished for the SVM classifier.

#### 4.1.2. Sampled Dataset

Table 3 shows the classification results for the second data treatment corresponding to a subsample of the original dataset. Note that the correct classification rate decreased for all combinations, obtaining the best results combining the data from both sensors and the two types of signals, in particular 54.0% for C4.5, 60.3% for SVM and 52.1% for KNN classifiers. In the case of NB, it also required the use of the accelerometer data to improve the classification, unlike the previous data treatment which only required the gyroscope data.

From the best results by classifier in this second evaluation, Table 4 shows the individual F-measure by activity. The best results using this metric were obtained from the SVM classifier (0.603), and although it was worse than the best results from the previous data treatment (0.625–0.696), it considerably reduced the dispersion value (σ); like the rest of the classifiers when using this dataset (σ < 0.143 for sampled data vs. σ > 0.270 for imbalanced data). Regarding the activities, the two that involved the use of stairs reduced their score, while the activities that involved the use of an incline improved it. Likewise, walking on level ground, the activity with the highest number of instances in the original dataset (at least five times more than the other activities), reduced its score by half for all classifiers.

#### 4.1.3. Augmented Dataset

Table 5 presents the classification results for the third data treatment corresponding to the dataset balanced using synthetic data. In this case, the models that combined information from both sensors and both signals improved their classification with respect to the previous data treatments. Additionally, from this treatment of the data, the best classifiers were built using C4.5 (81.2%), SVM (68.0%) and KNN (79.6%); for NB the best result was obtained with the unbalanced dataset (68.2%). The best global classifier so far was the one built with the C4.5 algorithm and the FD signal from both sensors.

From the best results by classifier in the last evaluation, Table 6 shows individual F-measures. In this case the activities that had the best performances were again going down and going up stairs, however this time the activity of walking on level ground scored a similar performance. In addition of scoring the best accuracy results, C4.5 has the best F-measure with the lowest dispersion among the activities.

#### 4.1.4. Classification Comparison

**Figure 7:**
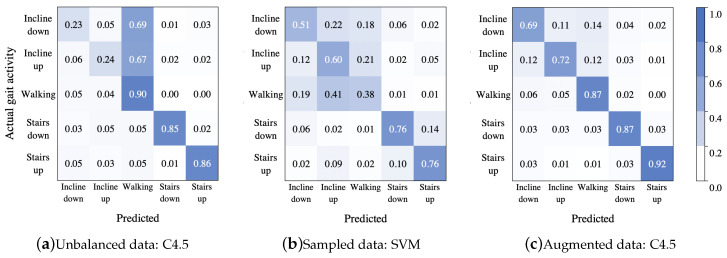
Confusion matrices for the best treatment by each classifier using unbalanced data from Table 2: C4.5 with features $Acc_{FD}+Gyr_{FD}$ (**a**), sampled data from Table 4: SVM with all features (**b**), and augmented data from Table 6: C4.5 with all features (**c**).

Finally, the best model for each data treatment was selected for comparing the inter-activity misclassification: (i) C4.5 built with features $Acc_{FD}+Gyr_{FD}$ (unbalanced data), (ii) SVM built with all features (sampled data), and (iii) C4.5 with all features (augmented data). Thus, to summarize the classification performance, a confusion matrix for each data treatment, was calculated. Figure 7 shows the confusion matrices from these classifiers, respectively. As it can be seen, the model in Figure 7a was overfitted to the majority class, because most instances of going down and going up an incline were misclassified as walking on the level ground, while the stairs activities presented the best proportion among sensitivity and specificity. In the second confusion matrix Figure 7b, there was a high confusion between the first three activities, and most instances of walking on the level ground were misclassified as going up an incline; in this case the stair activities presented confusion between them. The best classification is shown in Figure 7c, although it presented a minimum confusion in the first three activities.

### 4.2. Gait Classification Using Deep Learning

To test the CNN model (presented in Section 3.3.2) for the classification of the gait activity, the same scenarios presented in Section 4.1 are considered: (i) the unbalanced dataset, (ii) the sampled dataset, and (iii) the augmented dataset. The CNN model on each scenario was trained for 30 epochs. The metrics used to evaluate the performance of the CNN model in each case were precision, recall, accuracy and F-measure.

#### 4.2.1. Unbalanced Dataset

**Figure 8:**
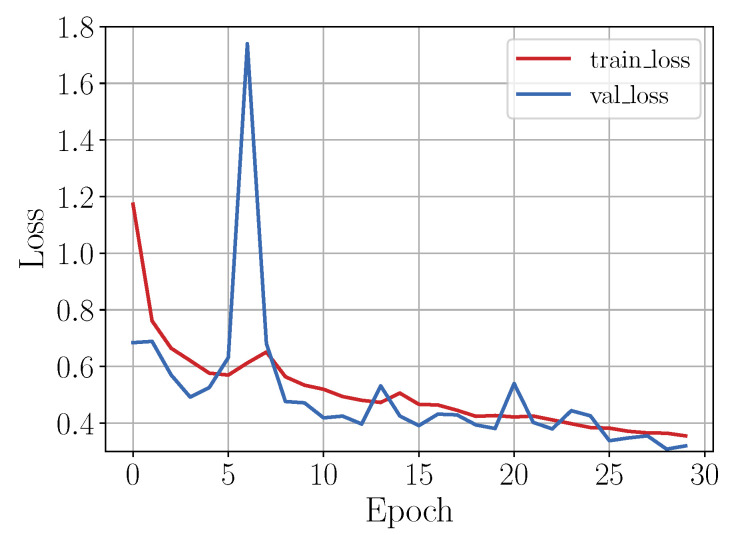
Loss behavior during training stage.

As in the shallow learning approach, 80% of the instances of each class of the OU-ISIR gait activities dataset was used to train the CNN model. The training instances were randomly and uniformly selected leaving the remaining ones for testing. Figure 8 shows the loss graph during the training process of the implemented CNN model. As can be seen in the figure, the exponential shape of the loss plot (red line) indicates that the model had a good learning rate and, given that the validation loss (blue line) remained close to the loss plot, it was then expected that the model trained does not present overfitting.

The classification report is presented in Table 7, where it can be seen that the overall accuracy of the model was around 89%. Furthermore, despite the fact that the CNN model was used on a dataset with unbalanced classes, it seems that the relevant features of each class were being captured in the training stage, which made it possible to have good classification performance, as shown by the F-measure.

#### 4.2.2. Sampled Dataset

A balanced dataset generated from the OU-ISIR gait activities dataset to train the model was used (80% instances for training and 20% for testing).

**Figure 9:**
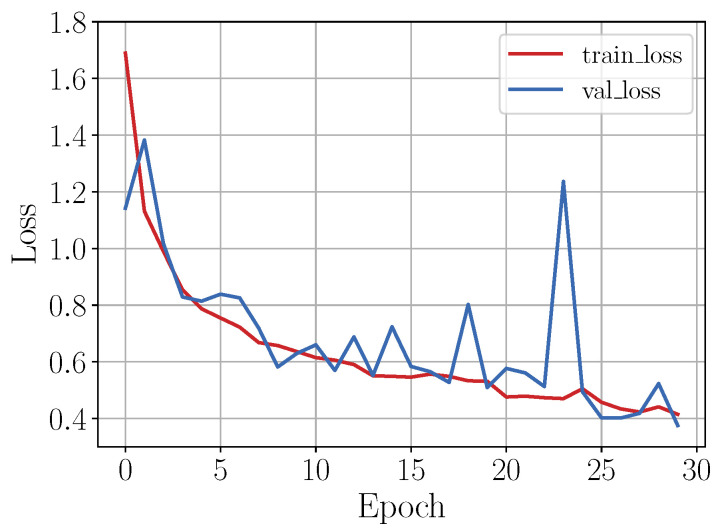
Loss behavior during training stage. To train the model the sampled dataset was used.

Figure 9 shows the loss curve during the training process. As in the previous case, the validation loss curve, as well as the loss curve, showed an exponential shape besides staying close, indicating that the model had a good learning rate.

Table 8 shows the classification report. Even though the number of instances on each class was lower (except for the minority class) than in the previous case, the accuracy remained almost the same. However, it can be observed that the F-measure for the walking class decreased. Possible reasons for this result are, on the one hand, that perhaps for this particular class it was necessary to have more data in the training stage, and on the other hand, that the data selected during sampling might not be representative enough for the class.

#### 4.2.3. Augmented Dataset

**Figure 10:**
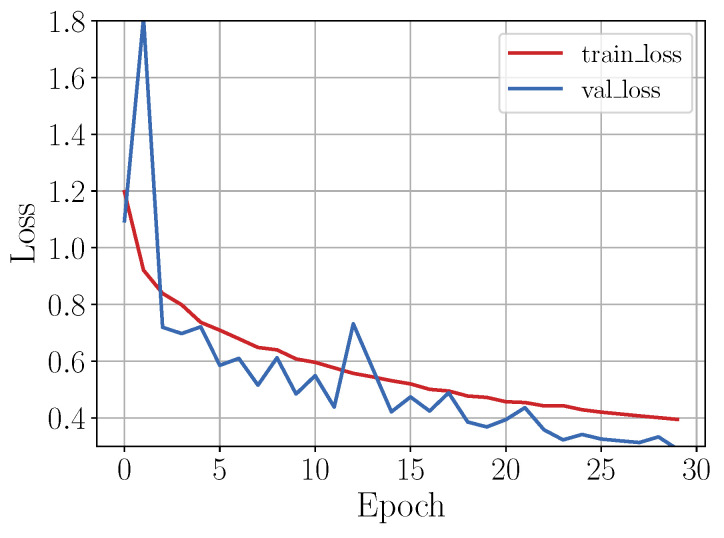
Loss behavior during training stage. To train the model the augmented dataset was used.

As a last case, the CNN model was trained using a dataset generated by means of data augmentation techniques. Thus, given that the amount of data per class was considerably larger than in the previous cases, it was expected that the learning rate of the network was good, as shown in Figure 10.

The performance of the trained model is summarized in Table 9. From this table, it can be concluded that the CNN model performance improved. For instance, the overall accuracy was almost 93%, a value that is 3% higher compared to the accuracy value of the first case and 2% higher compared to the second case.

#### 4.2.4. Classification Comparison

**Figure 11:**
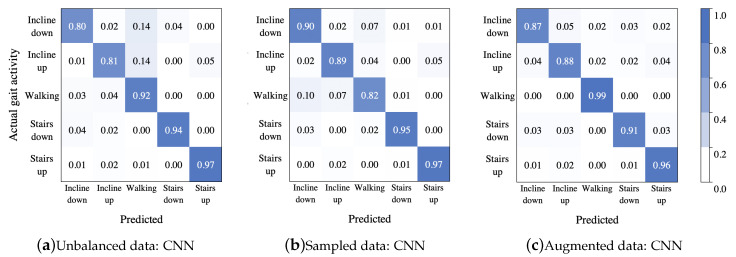
Confusion matrices showing the CNN model classification performance after being training by a unbalanced data, sampled data and augmented data.

The performance of each trained model can be summarized in the confusion matrices (in a normalized form) shown in Figure 11.

For the unbalanced dataset scenario, it can be observed from the confusion matrix shown in Figure 11a, that misclassified instances of incline down and incline up were predicted as walking (14% in each case). For the rest of the gait activities, the classification rate was over 90%. However, note that from the column sums, it can be concluded that the walking class was an attractor (more samples than expected are assigned to this class), since it had 21% above the expected value of samples assigned to it, while the incline down class was the strongest repeller (less samples than expected were assigned to this class) with 11% less samples assigned to it than expected. In contrast, although in the second case the dataset had less number of instances, the classification rate for the incline up and incline down classes improved, but the classification rate for the walking class decreased by around 10% (see Figure 11b); besides, there were no strong attractors or repellers, though the class incline down was the strongest attractor with 5% above expected value of samples assigned to it, and the class stairs down was the strongest repeller with 5% less samples assigned to it than expected. On the other hand, from results shown in Figure 11c, it can be seen that even though a large amount of data was generated by means of the data augmentation techniques, the CNN model performed well, even better than in the other scenarios.

F-measure was calculated to assess and compare the performance of the CNN model, trained from an unbalanced, sampled and augmented dataset.

Table 10 shows that the CNN model performed well regardless of the dataset used for training. Nevertheless, the best results were presented when the augmented dataset was used, which was expected due to the considerably greater amount of data, compared to the other datasets, used in the training process. However, the amount of time to train the CNN model was also considerably greater (about 30 h on a standard laptop with an Intel Core i5 processor and 16 GB of RAM). In contrast, the lowest performance was presented when the CNN model was trained with the unbalanced dataset, except for the walking class which had around 63% of the total number of instances of the entire unbalanced dataset.

### 4.3. Discussion

The classification results from the shallow learning approach vary significantly for all the combinations of features used, from 39.5% to 81.2%, either using balanced, sampled or augmented data. In general, the best results are obtained using the features extracted from the forward-direction signal FD of both accelerometer and gyroscope. There was no inference algorithm that would work properly for all three data treatments, however, the classifier built using C4.5 obtained the best overall result with this approach.

Regarding the results reported in the previous work in [15], in which only data from the accelerometer, with balanced classes, of seven young subjects were used, the correct classification average of the four classifiers from the combination $Acc_{FD}+Acc_{XYZ}$ was 69.4% (σ = 11.4), while in this study it was 65.7% (σ = 3.7) for the unbalanced data, 51.7% (σ = 2.7) for the sampled data, and 66.0% (σ = 8.0) for the augmented data. Although the average is close for data treatments 1 and 3, the inter-classifier dispersion value was considerably reduced for all three treatments. From the perspective of evaluating the proposed method with a much larger number of participants (745) with widely dispersed ages (2–78 years), it is worth mentioning that the results are satisfactory.

Nevertheless, as it is shown in the experimental results, the deep learning approach presents improvements in the gait activity classification task presented in this work, making it a good alternative to solve this kind of problems. Furthermore, encoding a time series as a color image, proved to be a good strategy to extract features through the CNN model training stage. The CNN is used to build a gait activities classifier, which showed to have a better performance than the so-called shallow learning methods. However, it is important to consider the training time which increases as the number of data increases, and in particular for the case of the augmented database it was around 30 h, nevertheless, this time can be improved if the training stage is performed using a GPU. Although it is a fact that training a CNN model is a slow process, the execution time is not related to it but with the input parameters instead, in this case the image size. The average classification time was computed for the largest dataset, resulting in a value around 0.016 s, making it feasible to use this classifier in real-time scenarios.

**Figure 12:**
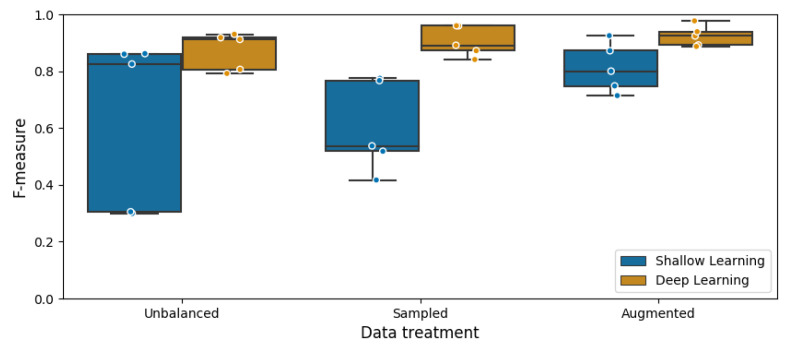
Comparison between conventional (shallow) and deep learning techniques.

The improvement of the deep learning approach with respect to the shallow learning based approach can be seen in Figure 12. This figure shows the F-score obtained by the best classifiers of the two approaches, using the three data treatments: unbalanced, sampled, and augmented. The classifiers selected from the shallow learning approach are described in Figure 7, while those based on deep learning were presented in Table 10 and are shown in Figure 11.

As it can be noticed, the measure of central tendency is superior for deep learning, and the measure of dispersion is less in all three treatments. Surprisingly, the same data trends relative to its counterpart for all proposed treatments can be seen. In the case of unbalanced data, in both approaches, the classifiers have good results for three of the classes, while for both, the incline-related ones, have poor results and far from the others, so the median of the boxplots is grouped at the top. In contrast, with the sampled data the classifiers of both approaches have the best results for stairs activities, grouping the other three activities down from the boxplot. In the third case, using augmented data, the results of the classifiers of both approaches, which are the best overall, are close for the five activities, however, the activity best classified for the deep approach was walking on level ground, while for the other approach it was going up stairs. The results of the last treatment could be explained by the generation of synthetic data, but in the deep approach the best activity was walking, from which no additional data were generated.

Using a deep learning-based approach to authenticate, label, recognize or even classify gait time series, has proven to be a better strategy, as in almost all cases it has shown improvements over the results obtained with the shallow approaches. For instance, in [25] a gender recognition as well as age recognition with around 88.6% of accuracy have been achieved. In works like the one presented in [24] where a multi-task approach is proposed, the authors present a solution for a gender and age recognition as well as for people authentication, obtaining up to 96% of identification accuracy. Then, for gait activities classification task (like the one presented in this work), a classification accuracy around 90% shows that deep learning approaches should be considered as a good choice for this task just as it was in the other tasks.

Finally, it is important to notice that when using unbalanced data, overfitted models are usually generated, particularly when the order is very high between the majority class and the rest of the classes. As can be seen in the results obtained from the proposed approaches, the use of balanced data improves the sensitivity as well as the specificity of the gait activity classifiers.

## 5. Conclusions

In the present work, gait activities were classified by using two computational learning approaches on a large-scale dataset. For the treatment of this dataset, three types of treatments were presented, one of them considering the unbalanced original data, and the other two by balancing the number of instances per class. Balancing by increasing data requires adapting synthetic data generation techniques to the problem domain, since the data was previously segmented by step size, which represented a detailed study of inertial data. The first learning approach consisted of adapting a previous method for recognizing gait activities, and although the same good results were not obtained, the best classification model (shallow approach) was able to properly fit the huge dataset. This characteristic led us to propose a second approach that could deal with a large amount of data, and it was solved by transforming inertial sensor signals into images to be used by a method based on deep learning. This second approach obtained very good results, even when using unbalanced data.

The dataset considered was collected using inertial sensors attached to the waist using a belt, allowing redundant data among the sensors. Since the images transformed from the sensor signals can be mixed or even grouped by changing their representation, it is feasible to use multiple sensors placed on different anatomical references to generate rich representations, either hierarchically or topologically, for example using inertial sensors embedded in activity trackers or smartwatches, or even placing the inertial sensors on the lower extremities.

Future work will investigate if the methods presented in this study can be used with gait data captured in naturalistic environments, in addition, to propose an online recognition approach, taking advantage of both approaches for different tasks, e.g., recognizing periods of ’activity’ and ’not activity’ using conventional classification, and the periods of activity classifying them into types of gait activity. Additionally, it is possible to focus the work on customized models or by type of population, e.g., children or elderly, adding a retraining stage from the general model of gait recognition and classification, using the data that are captured online.

## Figures and Tables

**Table 1:** Correctly classified instances (%) using unbalanced data, number of instances = 15,691.

Sensor	Signal	Features	NB	C4.5	SVM	KNN	Avg (σ)
	FD	5	66.0	68.5	69.1	59.5	65.8 (4.4)
Acc	XYZ	5	63.8	66.5	68.4	54.8	63.4 (6.0)
	FD+XYZ	9	63.7	67.8	69.6	61.6	65.7 (3.7)
	FD	5	**68.2**	71.9	71.8	60.4	68.1 (5.4)
Gyr	XYZ	5	63.0	66.3	68.2	54.2	62.9 (6.2)
	FD+XYZ	9	56.8	69.9	72.2	62.6	65.4 (7.0)
	FD	9	67.9	**72.5**	72.8	66.4	**69.9** (3.2)
Acc + Gyr	XYZ	9	61.3	65.2	69.4	57.7	63.4 (5.0)
	FD+XYZ	17	55.3	70.6	**73.2**	**69.7**	67.2 (8.1)

Acc: Accelerometer, Gyr: Gyroscope, NB: Naive Bayes, SVM: Support Vector Machines, KNN: K-Nearest Neighbors.

**Table 2:** F-measures for all gait activities using the best treatment by each classifier from unbalanced data (Table 1).

Gait Activity	NB	C4.5	SVM	KNN	Avg (σ)
Going down an incline	0.205	0.299	0.038	0.349	0.223 (0.137)
Going up an incline	0.231	0.305	0.007	0.315	0.215 (0.143)
Walking on level ground	0.795	0.825	0.827	0.795	0.811 (0.018)
Going down stairs	0.676	0.860	0.904	0.862	0.826 (0.102)
Going up stairs	0.833	0.862	0.912	0.893	0.875 (0.035)
Weighted avg	0.625	0.696	0.633	0.687	0.660 (0.087)
(σ)	(0.307)	(0.270)	(0.424)	(0.257)	

Treatments. NB: $Gyr_{FD}$ C4.5: $Acc_{FD}+Gyr_{FD}$ SVM and KNN: $Acc_{FD}+Acc_{XYZ}+Gyr_{FD}+Gyr_{XYZ}$.

**Table 3:** Correctly classified instances (%) using sampled data, number of instances = 4400.

Sensor	Signal	Features	NB	C4.5	SVM	KNN	Avg (σ)
	FD	5	51.7	52.0	53.6	47.0	51.1 (2.8)
Acc	XYZ	5	46.9	45.7	50.3	42.2	46.3 (3.3)
	FD+XYZ	9	50.0	51.8	55.5	49.7	51.7 (2.7)
	FD	5	46.0	43.2	47.4	40.0	44.2 (3.3)
Gyr	XYZ	5	45.3	44.3	48.0	39.5	44.3 (3.5)
	FD+XYZ	9	45.7	45.2	50.5	43.5	46.2 (3.0)
	FD	9	**53.2**	53.5	58.0	50.6	53.8 (3.1)
Acc + Gyr	XYZ	9	47.5	47.4	53.0	44.5	48.1 (3.6)
	FD+XYZ	17	50.0	**54.0**	**60.3**	**52.1**	**54.1** (4.4)

Acc: Accelerometer, Gyr: Gyroscope, NB: Naive Bayes, SVM: Support Vector Machines, KNN: K-Nearest Neighbors.

**Table 4:** F-measures for all gait activities using the best treatment by each classifier from sampled data (Table 3).

Gait Activity	NB	C4.5	SVM	KNN	Avg (σ)
Going down an incline	0.421	0.455	0.537	0.423	0.459 (0.137)
Going up an incline	0.498	0.444	0.518	0.422	0.471 (0.143)
Walking on level ground	0.373	0.402	0.417	0.404	0.399 (0.018)
Going down stairs	0.676	0.711	0.775	0.690	0.713 (0.102)
Going up stairs	0.671	0.691	0.767	0.690	0.705 (0.035)
Weighted avg	0.528	0.540	0.603	0.526	0.549 (0.041)
(σ)	(0.125)	(0.132)	(0.143)	(0.134)	

Treatments. NB: $Acc_{FD}+Gyr_{FD}$ C4.5, SVM and KNN: *Acc_FD_* + *Acc_XYZ_* + *Gyr_FD_* + *Gyr_XYZ_*.

**Table 5:** Correctly classified instances (%) using augmented data, number of instances = 49,325.

Sensor	Signal	Features	NB	C4.5	SVM	KNN	Avg (σ)
	FD	5	57.7	71.1	60.3	68.9	64.5 (6.5)
Acc	XYZ	5	51.9	60.7	57.3	57.0	56.7 (3.6)
	FD+XYZ	9	56.4	72.1	62.4	73.2	66.0 (8.0)
	FD	5	44.7	48.1	46.8	47.7	46.8 (1.5)
Gyr	XYZ	5	43.2	44.7	45.6	43.7	44.3 (1.1)
	FD+XYZ	9	43.2	51.3	49.6	57.2	50.3 (5.8)
	FD	9	**60.0**	**81.2**	65.1	77.2	70.9 (10.0)
Acc + Gyr	XYZ	9	55.5	66.0	62.5	67.1	62.8 (5.2)
	FD+XYZ	17	58.9	81.2	**68.0**	**79.6**	**71.9** (10.5)

Acc: Accelerometer, Gyr: Gyroscope, NB: Naive Bayes, SVM: Support Vector Machines, KNN: K-Nearest Neighbors.

**Table 6:** F-measures for all gait activities using the best treatment by each classifier from sampled data (Table 5).

Gait Activity	NB	C4.5	SVM	KNN	Avg (σ)
Going down an incline	0.325	0.714	0.511	0.674	0.556 (0.177)
Going up an incline	0.554	0.748	0.583	0.704	0.647 (0.093)
Walking on level ground	0.787	0.800	0.795	0.761	0.786 (0.017)
Going down stairs	0.552	0.872	0.700	0.899	0.756 (0.162)
Going up stairs	0.698	0.925	0.769	0.944	0.834 (0.120)
Weighted avg	0.583	0.812	0.672	0.797	0.716 (0.114)
(σ)	(0.157)	(0.078)	(0.109)	(0.107)	

Treatments. NB and C4.5: $Acc_{FD}+Gyr_{FD}$, SVM and KNN: *Acc_FD_* + *Acc_XYZ_* + *Gyr_FD_* + *Gyr_XYZ_*.

**Table 7:** Classification report of the CNN model trained with the unbalanced dataset.

Gait Activity	Precision	Recall	F-Measure	Support
Going down an incline	0.810	0.802	0.806	419
Going up an incline	0.773	0.812	0.792	377
Walking on level ground	0.942	0.919	0.930	1973
Going down stairs	0.883	0.943	0.912	176
Going up stairs	0.872	0.968	0.918	190
Accuracy			0.895	3135
Macro Avg.	0.856	0.889	0.871	3135
Weighted Avg.	0.896	0.895	0.895	3135

**Table 8:** Classification report of the CNN model trained with the sampled dataset.

Gait Activity	Precision	Recall	F-Measure	Support
Going down an incline	0.849	0.897	0.872	175
Going up an incline	0.891	0.891	0.891	175
Walking on level ground	0.867	0.817	0.841	175
Going down stairs	0.971	0.949	0.96	175
Going up stairs	0.95	0.971	0.96	175
Accuracy			0.905	875
Macro Avg.	0.905	0.905	0.905	875
Weighted Avg.	0.905	0.905	0.905	875

**Table 9:** Classification report of the CNN model trained with the augmented dataset.

Gait Activity	Precision	Recall	F-Measure	Support
Going down an incline	0.912	0.873	0.892	1553
Going up an incline	0.890	0.885	0.887	1594
Walking on level ground	0.964	0.989	0.977	1972
Going down stairs	0.935	0.914	0.924	1796
Going up stairs	0.922	0.956	0.939	1781
Accuracy			0.927	8696
Macro Avg.	0.925	0.923	0.924	8696
Weighted Avg.	0.927	0.927	0.927	8696

**Table 10:** F-measures for all gait activities using CNN model from unbalanced, sampled and augmented data.

	Data Treatment		
Gait Activity	Unbalanced	Sampled	Augmented
Going down an incline	0.806	0.872	0.892
Going up an incline	0.792	0.891	0.887
Walking on level ground	0.930	0.841	0.977
Going down stairs	0.912	0.960	0.924
Going up stairs	0.918	0.960	0.939
Weighted avg	0.895	0.905	0.927
(σ)	(0.064)	(0.048)	(0.033)

## Abbreviations

The following abbreviations are used in this manuscript:

DMM	Data Modifying the Magnitude of the signal’s
DMF	Data Modifying the Frequency of the signals
ACC	Acceleration sensor
GYR	Gyroscope sensor
FD	Forward Direction signal
XYZ	magnitude vector signal
NB	Naive Bayes
SVM	Support Vector Machines
KNN	K-Nearest Neighbors
CNN	Convolutional Neuronal Network
DL	Deep Learning
GAF	Gramian Angular Field

## References

[1] Lopez-Nava I.H., Muñoz-Meléndez A. (2019). Human action recognition based on low-and high-level data from wearable inertial sensors. Int. J. Distrib. Sens. Networks.

[2] López-Nava I.H., Muñoz-Meléndez A. (2018). High-level features for recognizing human actions in daily living environments using wearable sensors. Proceedings.

[3] Woznowski P., Kaleshi D., Oikonomou G., Craddock I. (2016). Classification and suitability of sensing technologies for activity recognition. Comput. Commun..

[4] Long X., Yin B., Aarts R.M. Single-accelerometer-based daily physical activity classification. Proceedings of the 2009 Annual International Conference of the IEEE Engineering in Medicine and Biology Society.

[5] Chandler J., Brazendale K., Beets M., Mealing B. (2016). Classification of physical activity intensities using a wrist-worn accelerometer in 8–12-year-old children. Pediatr. Obes..

[6] Duncan M.J., Rowlands A., Lawson C., Leddington Wright S., Hill M., Morris M., Eyre E., Tallis J. (2019). Using accelerometry to classify physical activity intensity in older adults: What is the optimal wear-site?. Eur. J. Sport Sci..

[7] Startzell J.K., Owens D.A., Mulfinger L.M., Cavanagh P.R. (2000). Stair negotiation in older people: A review. J. Am. Geriatr. Soc..

[8] Hall K.S., Hyde E.T., Bassett D.R., Carlson S.A., Carnethon M.R., Ekelund U., Evenson K.R., Galuska D.A., Kraus W.E., Lee I.M. (2020). Systematic review of the prospective association of daily step counts with risk of mortality, cardiovascular disease, and dysglycemia. Int. J. Behav. Nutr. Phys..

[9] Saint-Maurice P.F., Troiano R.P., Bassett D.R., Graubard B.I., Carlson S.A., Shiroma E.J., Fulton J.E., Matthews C.E. (2020). Association of daily step count and step intensity with mortality among US adults. JAMA.

[10] Goodfellow I., Bengio Y., Courville A. (2016). Deep Learning.

[11] LeCun Y., Bengio Y., Hinton G. (2015). Deep learning. Nature.

[12] Wan S., Qi L., Xu X., Tong C., Gu Z. (2019). Deep learning models for real-time human activity recognition with smartphones. Mob. Netw. Appl..

[13] Nguyen K.T., Portet F., Garbay C. Dealing with Imbalanced data sets for Human Activity Recognition using Mobile Phone sensors. Proceedings of the 3rd International Workshop on Smart Sensing Systems.

[14] He H., Garcia E.A. (2009). Learning from imbalanced data. IEEE Trans. Knowl. Data Eng..

[15] Lopez-Nava I.H., Garcia-Constantino M., Favela J. (2019). Recognition of Gait Activities Using Acceleration Data from A Smartphone and A Wearable Device. Proceedings.

[16] Guiry J.J., van de Ven P., Nelson J., Warmerdam L., Riper H. (2014). Activity recognition with smartphone support. Med. Eng. Phys..

[17] Wei Z., Qinghui W., Muqing D., Yiqi L. A new inertial sensor-based gait recognition method via deterministic learning. Proceedings of the 2015 34th Chinese Control Conference (CCC).

[18] Sprager S., Juric M.B. (2015). An efficient HOS-based gait authentication of accelerometer data. IEEE Trans. Inf. Forenic Sec..

[19] Subramanian R., Sarkar S., Labrador M., Contino K., Eggert C., Javed O., Zhu J., Cheng H. Orientation invariant gait matching algorithm based on the Kabsch alignment. Proceedings of the IEEE International Conference on Identity, Security and Behavior Analysis (ISBA 2015).

[20] Caldas R., Hu Y., de Lima Neto F.B., Markert B. Self-organizing maps and fuzzy c-means algorithms on gait analysis based on inertial sensors data. Proceedings of the International Conference on Intelligent Systems Design and Applications.

[21] Lee S.M., Yoon S.M., Cho H. Human activity recognition from accelerometer data using Convolutional Neural Network. Proceedings of the IEEE International Conference on Big Data and Smart Computing (BigComp).

[22] Lin H.W., Tegmark M., Rolnick D. (2017). Why does deep and cheap learning work so well?. J. Stat. Phys..

[23] Nguyen K.T., Vo-Tran T.L., Dinh D.T., Tran M.T. Gait recognition with multi-region size convolutional neural network for authentication with wearable sensors. Proceedings of the International Conference on Future Data and Security Engineering.

[24] Delgado-Escano R., Castro F.M., Cózar J.R., Marín-Jiménez M.J., Guil N. (2018). An end-to-end multi-task and fusion CNN for inertial-based gait recognition. IEEE Access.

[25] Sun Y., Lo F.P.W., Lo B. A Deep Learning Approach on Gender and Age Recognition using a Single Inertial Sensor. Proceedings of the 2019 IEEE 16th International Conference on Wearable and Implantable Body Sensor Networks (BSN).

[26] Ahad M.A.R., Ngo T.T., Antar A.D., Ahmed M., Hossain T., Muramatsu D., Makihara Y., Inoue S., Yagi Y. (2020). Wearable Sensor-Based Gait Analysis for Age and Gender Estimation. Sensors.

[27] Ngo T.T., Makihara Y., Nagahara H., Mukaigawa Y., Yagi Y. (2014). The largest inertial sensor-based gait database and performance evaluation of gait-based personal authentication. Pattern. Recogn..

[28] Um T.T., Pfister F.M., Pichler D., Endo S., Lang M., Hirche S., Fietzek U., Kulić D. Data augmentation of wearable sensor data for parkinson’s disease monitoring using convolutional neural networks. Proceedings of the 19th ACM International Conference on Multimodal Interaction.

[29] Steven Eyobu O., Han D.S. (2018). Feature representation and data augmentation for human activity classification based on wearable IMU sensor data using a deep LSTM neural network. Sensors.

[30] Friedman N., Geiger D., Goldszmidt M. (1997). Bayesian network classifiers. Mach. Learn..

[31] Quinlan J.R. (2014). C4.5: Programs for Machine Learning.

[32] Cortes C., Vapnik V. (1995). Support-vector networks. Mach. Learn..

[33] Keller J.M., Gray M.R., Givens J.A. (1985). A fuzzy k-nearest neighbor algorithm. IEEE Trans. Syst. Man. Cybern..

[34] Sengupta S., Basak S., Saikia P., Paul S., Tsalavoutis V., Atiah F., Ravi V., Peters R.A. (2020). A Review of Deep Learning with Special Emphasis on Architectures, Applications and Recent Trends. Knowl.-Based Syst..

[35] Hamme T., Garofalo G., Argones Rúa E., Preuveneers D., Joosen W. (2019). A Systematic Comparison of Age and Gender Prediction on IMU Sensor-Based Gait Traces. Sensors.

[36] Zhao Y., Zhou S. (2017). Wearable Device-Based Gait Recognition Using Angle Embedded Gait Dynamic Images and a Convolutional Neural Network. Sensors.

[37] Wang Z., Oates T. Imaging Time-Series to Improve Classification and Imputation. Proceedings of the 24th International Conference on Artificial Intelligence.

[38] Nair V., Hinton G.E. Rectified Linear Units Improve Restricted Boltzmann Machines. Proceedings of the 27th International Conference on International Conference on Machine Learning.

